# Production and Characterization of Pectinase Through Solid-State Fermentation of Orange Peels by a Mutant Yeast Strain

**DOI:** 10.1155/ijfo/8853746

**Published:** 2025-03-07

**Authors:** Uroosa Ejaz, Asma Hanif, Ahsan Ali Khan, Laiba Jawad, Isha Rasheed, Bushra Noor, Amal S. Alswat, Muhammad Sohail

**Affiliations:** ^1^Department of Biosciences, SZABIST University, Karachi, Pakistan; ^2^Department of Microbiology, University of Karachi, Karachi, Pakistan; ^3^Department of Biotechnology, Taif University, Taif, Saudi Arabia

**Keywords:** central composite design, characterization, mutant, orange peels, pectinase, yeast

## Abstract

Pectinases hydrolyze pectin in plant biomass and are commonly used in the fruit juice industry. In this study, pectinase produced by a mutant yeast strain, *Geotrichum candidum* AHC1, was characterized and used to clarify the orange juice. The strain AHC1 produced 76.08 IU mL^−1^ pectinase after fermenting orange peel powder under solid-state conditions. This yield was several folds higher than *G. candidum* AA15 (wild type) and *Saccharomyces cerevisiae* MK-157. Moreover, *S. cerevisiae* MK-157, *G. candidum* AA15, and *G. candidum* AHC1 produced 42.62, 58.28, and 75.28 IU mL^−1^ pectinase, respectively, under submerged fermentation. Consequently, pectinase from AHC1 was characterized by using a central composite design. Results indicated that pectinase from AHC1 exhibited maximum activity at 35°C, 16.47 min reaction time, 1.89% substrate concentration, and pH 5.4. Under optimized conditions, the preparation exhibited 87 IU mL^−1^ pectinase activity, which was correlated with the predicted value (95.13 IU mL^−1^). Furthermore, *K*_*m*_ of the crude pectinase (16 mg mL^−1^) was found to be greater in comparison to the purified pectinase, demonstrating its high affinity to pectin. The treatment of orange juice with pectinase resulted in increased yield and clarity within 30 min. This study provides prospects for a citrus circular bioeconomy by utilizing orange peels to produce pectinase and subsequently using pectinase to clarify orange juice.

## 1. Introduction

In the past few decades, the demand for enzymes in the food industry has grown extensively owing to health awareness in the general public and changing lifestyles. Production of good-quality juice is not possible without using enzymes. Juices obtained through mechanical grinding or crushing of fruits resulted in jelly-like pulp due to the presence of pectin, thus making the juice more viscous [1]. Depending on the type of fruit juice, enzymatic treatment is given to degrade complex pectin structure and to make the product according to the consumers' acceptance for complete clearance of haze and turbidity and decreased viscosity [2]. Pectinase treatment of juice enhances the yield and flavor [3].

For large-scale applications, pectinase production needs to be improved, which is generally carried out by using some inducers such as purified pectin or crude agroindustrial residue, including orange peels, in the microbiological medium [4–6]. Citrus peels are abundant waste worldwide, as citrus fruit is one of the largest fruit crops, with 100 million metric tons of annual production. Oranges alone account for 68 million tons, making up 8.5% of the world's total fruit production [7]. Consequently, orange peels can serve as a promising substrate for the production of pectinase. Orange peels contain 25% pectin, 22% cellulose, 11% hemicellulose, and 23% soluble sugars [8]. This composition is considered promising for the growth of microorganisms. Orange peels can also serve as a substrate under solid-state conditions, which offer several advantages such as low cost, high yield, and less chance of contamination [9].

Plants and various microbes, including molds, bacteria, and yeasts, are known to produce pectinase. A major proportion of food enzymes are of fungal origin, particularly from a filamentous fungus, *Aspergillus niger* [10]; however, fungi produce a heterogeneous mixture of pectinase. Yeasts offer several benefits over fungi, as they generally produce a single type of pectin-degrading enzyme; moreover, the shorter generation time of yeast renders the enzyme production process more economical by producing high titers in a shorter time [11]. However, Abd El-Aziz et al. [12] noticed that the use of yeasts for pectinase production has not been frequently reported. Yet, some strains of *Geotrichum* and *Saccharomyces* have been reported for their pectinolytic potential [13]. To improve pectinase production by yeasts, random mutagenesis has been identified as a promising approach considering the simple cellular and genetic organization of yeast cells compared to fungi. Indeed, physical or chemical mutagenesis has been previously used to increase the yield of yeast enzymes. In the context of pectinase production, Abd El-Aziz et al. [12] used mutagens, including ethidium bromide and UV rays, to mutate *Rhodotorula mucilaginosa* and obtained a high yield of pectinase, while Hanif et al. [14] mutagenized *Geotrichum candidum* AA15 and obtained a mutant strain that produced > 2-fold greater quantity of pectinase in contrast to the wild-type strain.

The overproduction of pectinase by the mutant or wild-type strain facilitates the application of this enzyme in fruit and vegetable processing. Indeed, the application of enzymes with anticipated characteristics and economical production for commercial applications has always been considered an essential aspect of research [15]. In order to obtain the desired product, process parameters need to be optimized, as enzymatic catalysis depends on various physicochemical properties, including the pH of the solution, reaction temperature, enzyme treatment time, and substrate concentration. Earlier, researchers adopted a conventional one-factor-at-a-time approach to optimize enzyme reaction [16]; however, a central composite design (CCD) can be opted for the characterization of enzymes. CCD is a statistical tool in which factors are optimized by investigating their five levels in statistically designed experiments [17].

In this study, the pectinolytic potential of a mutant derived from the *G. candidum* AA15 was evaluated. The strain AA15 was originally isolated from a mayonnaise sample by Ahmed et al. [11]. The results were also compared with the pectinase production by *Saccharomyces cerevisiae* MK-157 which was isolated from grapes [18]. Later, Hanif et al. [14] improved the strain *G. candidum* AA15 using UV and ethidium bromide and obtained a mutant strain, *G. candidum* AHC1. In this study, orange peels were used as a substrate to produce pectinase under solid-state and submerged fermentation. Pectinase produced by the mutant strain was characterized using CCD, and enzyme kinetic parameters were determined. Subsequently, the pectinase from the mutant strain was applied for orange juice clarification to illustrate the concept of circular bioeconomy.

## 2. Methodology

### 2.1. Preparation of Orange Peel Powder

Orange peels were locally collected and dried at 45°C for 72 h. Powder of orange peels of 100 *μ* particle size was obtained by using a grinder and sieve.

### 2.2. Revival of Yeast Culture and Inoculum Preparation

*G. candidum* AA15, *G. candidum* AHC1 [14], and *S. cerevisiae* MK-157 [13] were procured from the microbiology lab of the University of Karachi. Cultures were retrieved on Sabouraud's dextrose agar (Oxoid, United States) and kept at 30°C for 48 h. All the cultures were separately inoculated in Sabouraud's dextrose broth (Oxoid, United States) and kept at 30°C for 48 h. After incubation, the density of the inoculum was set to 1.0 OD_650_ using a Life Science UV/Vis Spectrophotometer, DU 730 (Sr/No. 1238553), Beckman-Coulter, United States, to maintain the uniformity of cultures.

### 2.3. Submerged Fermentation of Orange Peel

Mineral salt media (MSM) was prepared by mixing 10 mL of 10X solution A (2% Tween 80, 1% peptone, 0.1% CaCl_2_, 0.3% MgSO_4_.7H_2_O, 1.4% (NH_4_)_2_SO_4_, and 2% KH_2_PO_4_) and 90 mL of 100X solution B (0.29% CoCl_2_.6H_2_O, 0.14% ZnSO_4_.7H_2_O, 0.16% MnSO_4_.H_2_O, and 0.5% FeSO_4_.7H_2_O [16]. Inoculum (10%, *v*/*v*) was transferred in MSM, which contained 1% (*w*/*v*) orange peel powder [13], and incubated at 30°C for 48 h. After incubation, the contents were centrifuged at 2500 × *g* for 15 min, and the supernatant was saved as a crude pectinase preparation.

### 2.4. Solid-State Fermentation of Orange Peel

Inoculum and moistening agent (MSA) were added to 1 g of orange peel powder. Moisture was maintained at 80%. The contents were incubated for 48 h at 30°C. Post-incubation, 10 mL of pH 4.8 (50 mM Na citrate) buffer with 0.05% (*v*/*v*) Tween 80 was added to the media. It was then kept in a shaker at 150 rpm for 60 min. After 60 min, the content of the flask was filtered. Filtrate was centrifuged at 2500 × *g* for 15 min, and the supernatant was saved as a crude pectinase preparation.

### 2.5. Pectinase Assay

One unit (IU mL^−1^) of pectinase was defined as the amount of pectinase that can release 1 *μ*M of galacturonic acid under standard assay conditions. Commercial pectin (Sigma, United States) was used to prepare a 0.5% (*w*/*v*) substrate in 50 mM sodium citrate buffer (pH 4.8). Crude pectinase (25 *μ*L) was mixed with an equal volume of pectin and kept at 37°C for 15 min. After the reaction time, DNS reagent (150 *μ*L) was added, and the solution was boiled for 5 min, chilled on ice, and then 720 *μ*L distilled water was added. Absorbance was noted at 540 nm. A standard curve of galacturonic acid was used to calculate the amount of reducing sugars, and then the following formula was used to calculate pectinase units [13]:

 $$\begin{array}{c} \text{Pectinase }\left(\text{IU}/\text{mL}\right)=x\text{ mg of galacturonic acid}\times 7.403 \end{array}$$

### 2.6. Substrate Analysis

Native orange peel powder and fermented samples by *G. candidum* AHC1 were separately dried and analyzed by Fourier transform infrared spectroscopy and scanning electron microscopy using JASCO FTIR-4200 and Analytical Scanning Electron Microscope, JSM-6380 A, JEOL United States, respectively.

### 2.7. Characterization of Pectinase

Pectinase was extracted from the AHC1 strain grown on orange peels, and the pectinase activity was characterized by performing various experiments as suggested by the CCD using Minitab 18 software. The variables included reaction time, temperature, substrate concentration, and pH (Table S1). Furthermore, a response optimization experiment was generated by software after the analysis of CCD results.

### 2.8. Kinetic Study of Pectinase

Pectinase was purified as previously reported by Hanif et al. [14]. Crude pectinase was lyophilized in a freeze dryer (Trio Science Co., Model – TR-FDBT-50) at −40°C and 100 torr for 1 h. Lyophilized pectinase was resuspended in sodium citrate buffer (50 mM, pH 4.8) to the concentration of 0.1 g mL^−1^. Protein estimation of lyophilized pectinase was performed by the Bradford method using bovine serum albumin (BSA) as a standard [19]. Lyophilized pectinase (600 *μ*g) was loaded on a Sephadex G-100 column equilibrated with 20 mM sodium acetate buffer with pH 5.0. The same buffer was used to elute the protein fractions, and eluted peaks were monitored at 280 nm. Fractions were assayed for protein estimation and pectinase activity. Purified pectinase showed 3.85-fold purification with a specific activity of 119 U mg^−1^ and 13.93% of the pectinase yield. The Michaelis–Menten constant (*K*_*m*_) and maximum velocity (*V*_max_) of pectinase were estimated by conducting the pectinase assay in the presence of various concentrations of substrate ranging from 0.5% to 2.5%.

### 2.9. Pectinase Treatment of Orange Juice

Fresh orange juice was collected from a local fruit shop. Orange juice (20 mL) and pectinase (2 IU mL^−1^) were mixed. Commercial pectinase and deionized water were used as a positive and negative control, respectively. Samples were kept for 30 min at 35°C. After the reaction time, juice yield, juice turbidity, and pectin degradation tests were performed.

### 2.10. Juice Yield

Orange juice yield after pectinase treatment was determined using the following equation [20]:

 $$\begin{array}{c} \text{Orange juice yield }\left(\%\right)=\frac{\text{Amount of clear orange juice}}{\text{Amount of pulp}}\times 100 \end{array}$$

### 2.11. Measurement of Juice Turbidity

Turbidity of orange juice (10 mL) was measured by taking the optical density at 660 nm. Orange juice was centrifuged at 2500 × *g* for 15 min, and the absorbance of the supernatant was recorded. The resistance to clarification (cloud stability or turbidity) was deduced from the relative turbidity [21]:

 $$\begin{array}{c} \text{Relative turbidity }\left(\%\right)=\frac{\text{Turbidity after centrifugation}}{\text{Turbidity before centrifugation }}\times 100 \end{array}$$

### 2.12. Fruit Juice Clarity Analysis by Performing Pectin Degradation Test

Absolute ethanol (99 mL) was mixed with 1 mL of concentrated HCl to prepare acidified ethanol. Acidified ethanol (1 mL) was mixed with 0.5 mL of juice sample. Flocculation or clarification of fruit juice was visually observed.

### 2.13. Statistical Analysis

All the experiments were performed in triplicate, and result values were expressed in the form of a mean with insignificant standard deviation. CCD was made and analyzed by the software Minitab 18.

## 3. Results and Discussion

### 3.1. Production of Pectinase

**Figure 1:**
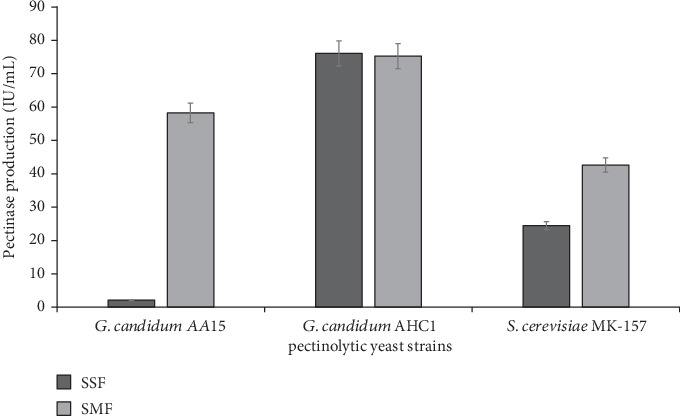
Production of pectinase using yeast strains (*G. candidum* AA15, *G. candidum* AHC1, and *S. cerevisiae* MK-157) by fermenting orange peels under solid state (SSF) and submerged (SMF) fermentation.

Pectinases find wide application in different industrial processes, and hence, cost-effective pectinase production is necessarily required. Therefore, in this study, orange peels were utilized as a crude substrate for the growth of pectinolytic yeasts. The mutant yeast strain of *G. candidum* (AHC1) produced the highest amount of pectinase (76.08 IU mL^−1^) by using orange peels under solid-state fermentation. In contrast, wild-type pectinolytic yeast strains, *G. candidum* AA15 and *S. cerevisiae* MK-157, produced 2.09 and 24.47 IU mL^−1^ pectinase, respectively, under similar conditions (Figure 1). Strain improvement through random mutagenesis has proven to be an effective strategy for the cost-efficient production of commercially important pectinase [14]. Moreover, solid-state fermentation of agrowaste further contributes to the reduction of production costs and offers many other advantages [22]. On the other hand, submerged fermentation of orange peels yielded 58.28, 75.28, and 42.62 IU mL^−1^ pectinase by *G. candidum* AA15, *G. candidum* AHC1, and *S. cerevisiae* MK-157, respectively (Figure 1). Previously, wild-type *G. candidum* AA15 produced 6.41 IU mL^−1^ pectinase, whereas the mutant *G. candidum* AHC1 yielded 12.03 IU mL^−1^ pectinase by utilizing commercial citrus pectin [14], while corncob-immobilized *S. cerevisiae* MK-157 produced 13.45 IU mL^−1^ pectinase under submerged fermentation [13]. Although molds have been frequently described for pectinase production [23], the use of yeasts is advantageous as they do not produce mycotoxins [24, 25]. Indeed, some of the yeast strains, including *Pichia anomala* AUMC 2674, *Pichia guilliermondii* AUMC 2663, and *Candida krusei* AUMC 8161, have been reported to control the mycotoxin production by 11 toxic fungal strains [26]. Although *A. niger* has also been reported for pectinase production, it requires more time to grow [27].

### 3.2. Analysis of Fermented Substrate

**Figure 2:**
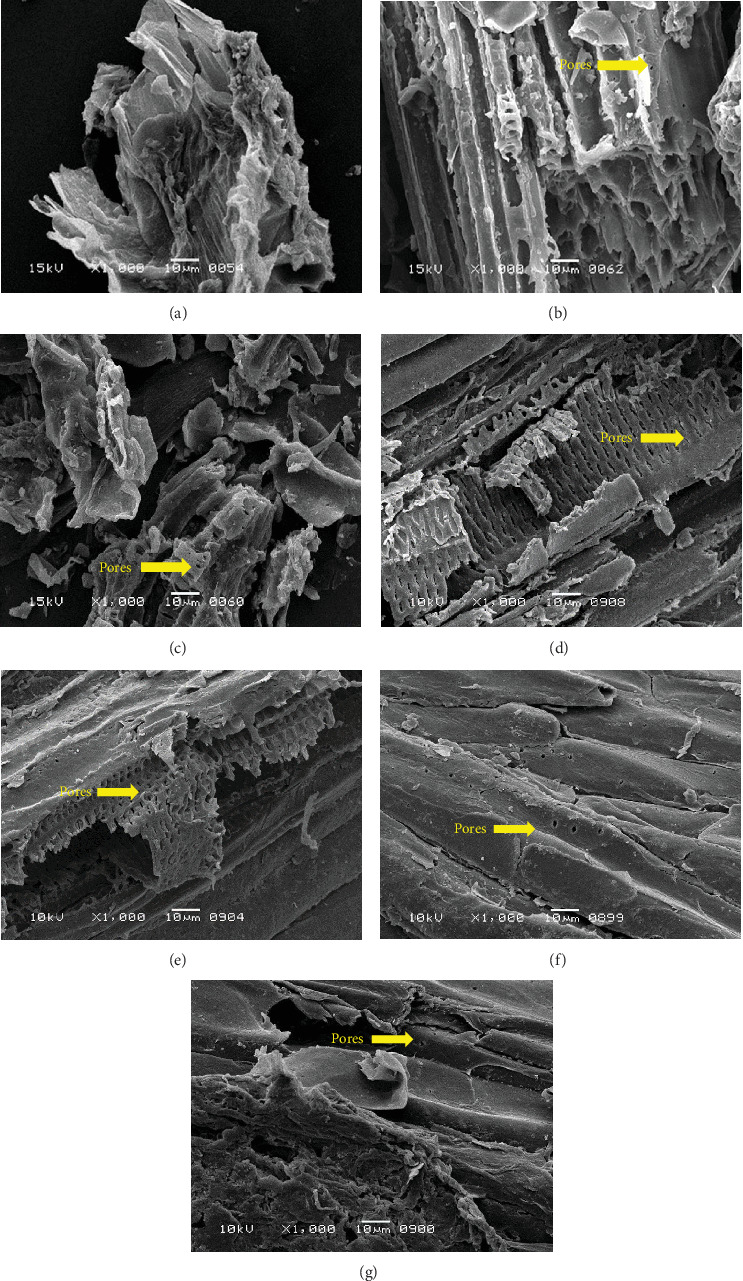
Scanning electron microscopic images of orange peel powder under different conditions. (a) Native, (b) fermented by *G. candidum* AA15 under solid-state condition, (c) fermented by *G. candidum* AA15 under submerged condition, (d) fermented by *G. candidum* AHC1 under solid-state condition, (e) fermented by *G. candidum* AHC1 under submerged condition, (f) fermented by *S. cerevisiae* MK-157 under solid-state condition, and (g) fermented by *S. cerevisiae* MK-157 under submerged condition.

The surface structure of native or unfermented orange peels clearly exhibited a dense and compact pattern under scanning electron microscopy (Figure 2) [28]. Fermentation of orange peels by yeast strains caused its destruction, and it became porous (Figures 2, 2, 2, 2, 2, and 2). Specifically, the structure of orange peels fermented by *G. candidum* AHC1 under submerged and solid-state conditions was cracked, sparse, and porous. It was distinctly observed that the orange peels fermented by *S. cerevisiae* MK-157 and *G. candidum* AA15 consisted of large damaged fibers. These morphological changes can be attributed to the removal of pectin content by the action of pectinase. Many researchers also reported the damaged structure of plant biomass due to hydrolysis by plant cell wall degrading enzymes [29].

**Figure 3:**
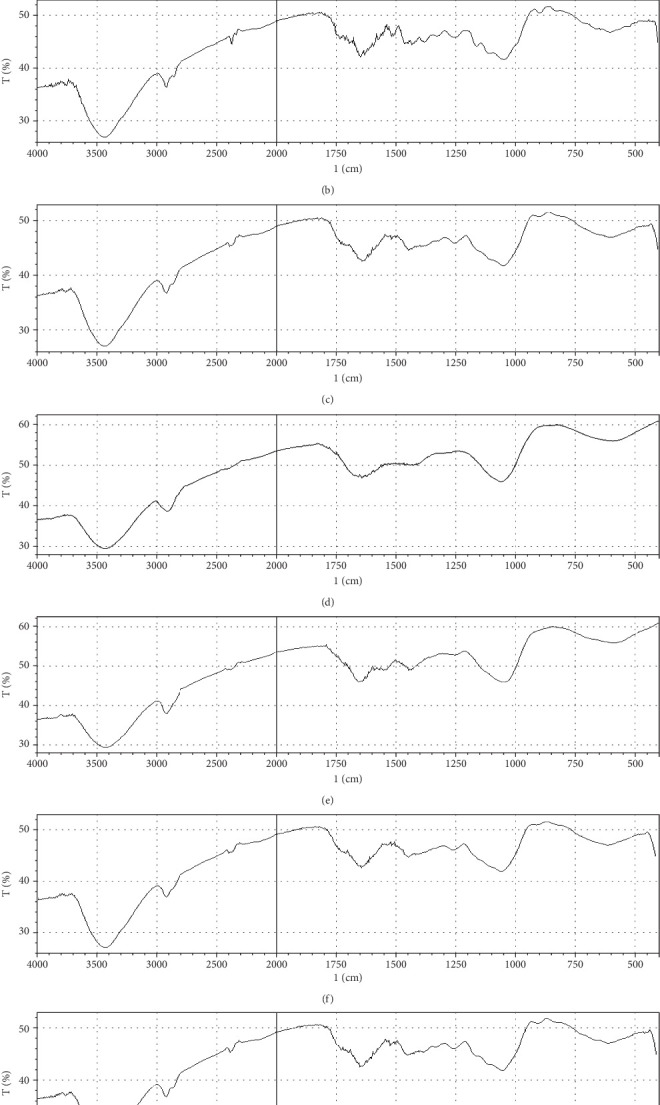
Fourier transform infrared spectroscopy images of orange peel powder under different conditions. (a) Native, (b) fermented by *G. candidum* AA15 under solid-state condition, (c) fermented by *G. candidum* AA15 under submerged condition, (d) fermented by *G. candidum* AHC1 under solid-state condition, (e) fermented by *G. candidum* AHC1 under submerged condition, (f) fermented by *S. cerevisiae* MK-157 under solid-state condition, and (g) fermented by *S. cerevisiae* MK-157 under submerged condition.

Fermentation processes can alter the chemical composition of plant materials; therefore, FTIR spectra of the native and fermented orange peels were obtained (Figure 3). The peaks between 1000 and 1700 cm^−1^ were of lignin. Peaks from 1550 to 1600 cm^−1^ were correlated to aromatic structure and 1350 cm^−1^ to the phenolic hydroxyl group. Peaks around 1050 cm^−1^ were assigned to the C–OH group of hemicellulose or the OH group of the lignin moiety [30]. Furthermore, the peaks at 1750 cm^−1^ reflected the C=O stretching band, which represented nonionic carboxy groups (i.e., –COOCH_3_ and –COOH) [31]. However, peaks around the region of 2900 cm^−1^ were due to symmetric and asymmetric C–H stretching vibrations presented in aliphatic acids. Particularly, the intense and broad absorption peaks around 3100 cm^−1^ are assigned to O–H stretching vibrations, which showed intramolecular and intermolecular hydrogen bonds of polymeric compounds [30]. Changes in FTIR spectra of fermented substrate clearly indicated the effect of fermentation on the composition of orange peel.

### 3.3. Characterization of Pectinase

**Figure 4:**
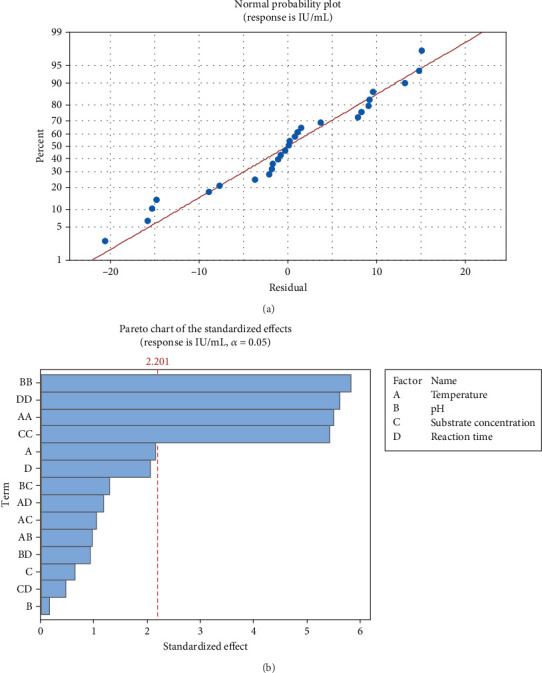
Analysis of central composite design. (a) Normal effect of factors on pectinase activity of *G. candidum* AHC1. (b) Pareto chart showing effect of factors on pectinase activity of *G. candidum* AHC1.

As the pectinase yield from the mutant yeast strain, *G. candidum* AHC1, was higher, therefore, pectinase from this organism was selected for characterization. Factors such as reaction time, temperature, pH, and substrate concentration usually influence the activity of enzymes. Temperature is an important factor, which can influence enzyme activity in two ways: directly influencing the reaction rate or by thermal denaturation of the enzyme. Moreover, a change in the pH of the reaction mixture can cause reversible and irreversible effects on the activity of enzymes. At a pH other than the optimum pH, amino acids of the enzyme molecules are ionized differently in a way that the enzyme cannot bind to the substrate as the active site loses its complementary structure [32]. Reaction time is also one of the critical parameters, which can affect the enzyme reaction rate. Depletion of substrate or denaturation of enzyme can occur if reaction time is prolonged [33]. It is also important to optimize the concentration of the substrate for an enzymatic reaction. An increase in substrate concentration can result in saturation of the active sites of the enzyme [3]. In the experiments performed according to the CCD, the pectinase activity of *G. candidum* AHC1 varied from 1.2 to 77.1 IU mL^−1^ (Table 1). The significance of the design was revealed by the *p* and *F* values of 0.006 and 4.88, respectively, in the ANOVA results (Table 2) with an *R*^2^ value of 0.86, which confirmed the validity of the model (Table 2). The effect of factors on pectinase activity was determined using a normal plot and Pareto chart (Figure 4). The normal plot represented an almost straight line of the points, which showed that variation in pectinase response is minimal (Figure 4). The Pareto chart revealed that the one-way interaction of substrate concentration, pH, reaction time, and temperature was significant (Figure 4). Moreover, the significance level (denoted by *α*) affects the reference line for statistical significance as described by Westcott [34]. One-way interaction of temperature, pH, substrate concentration, and reaction time was found to be significant (Figure 4). However, the directionality of the effect could not be determined by the Pareto chart. Therefore, coefficient values were checked to study the magnitude and direction of the factors (Table 3). Reaction time, the interaction of reaction time and substrate concentration, the interaction of pH and reaction time, and the interaction of temperature and substrate concentration showed a negative value of the coefficient, whereas all the other coefficient values were positive (Table 3). According to Ahmadi et al. [35], a positive coefficient denotes an increasing trend in the mean value of the response with an increase in the factors. Furthermore, the precision of the estimate of the coefficient was measured by calculating the standard error of the coefficient (SE Coef) [36]. A small SE Coef was observed for all the factors, which showed that the estimate of Coef was precise (Table 3). The regression equation was used to generate the contour factors (Figure S1). The interaction of temperature with reaction time and pH was observed to be inversely proportional to each other, whereas a directly proportional relation was found between the interaction of substrate concentration and temperature (Figure S1).

The response optimizer tool was used to optimize the pectinase activity. An optimal level was achieved, where composite desirability obtained its maximum value [37]. Results showed that the pectinase of mutant *G. candidum* AHC1 exhibited peak activity (87 IU mL^−1^) under the conditions of a reaction time of 16.47 min, a substrate concentration of 1.89%, a pH of 5.4, and a temperature of 35°C. This result was 91.45% close to the predicted value (95.13 IU mL^−1^). Previous research on the wild-type *G. candidum* AA15 indicated that the maximum pectinase activity was obtained at 35°C with a 25 min reaction time in an acidic environment at pH 5, with a 2.5% substrate concentration [16]. Hence, this study confirms that the pectinase of a mutant strain achieves maximum pectinase activity in a shorter reaction time and with lower substrate concentration.

### 3.4. Enzyme Kinetic Parameters

Evaluation of the kinetic parameters of an enzyme provides some understanding of its catalytic efficiency and mechanism [38]. The results of enzyme kinetics showed *V*_max_ and *K*_*m*_ of crude pectinase from the wild type (AA15) were 0.91 *μ*M min^−1^ and 24.69 mg mL^−1^, respectively, while the crude pectinase derived from the mutant yeast strain, AHC1, exhibited *V*_max_ and *K*_*m*_ of 6 *μ*M min^−1^ and 16 mg mL^−1^, respectively. However, *V*_max_ and *K*_*m*_ of AHC1-derived purified pectinase were 2.46 *μ*M min^−1^ and 10 mg mL^−1^, respectively (Table 4). *V*_max_ represents the maximum velocity at which the enzyme operates most effectively at a given substrate concentration, whereas *K*_*m*_ is the substrate concentration at which the enzyme works at half of *V*_max_ [39]. The *K*_*m*_ values for the crude enzymes (AA15 and AHC1) were higher (24.69 and 16 mg mL^−1^, respectively) compared to the purified pectinase (10 mg mL^−1^), indicating an increased substrate affinity of the purified enzyme. Hence, the purification of the enzyme resulted in increased affinity towards the substrate. The strong affinity of pectinase to its substrate makes it a cost-effective option for its use in industrial processes [40]. Similarly, Jalil and Ibrahim [41] reported that the crude pectinase isolated from *A. niger* LFP-1 had a *K*_*m*_ of 3.89 mg mL^−1^, claiming it as the highest substrate affinity. Gummadi and Panda [42] reported significant variations in *V*_max_ and *K*_*m*_ values among pectinases produced by different microorganisms. The kinetic parameters of the pectinase from AHC1 were comparable to the literature reports.

### 3.5. Pretreatment of Orange Juice by Pectinase

**Figure 5:**
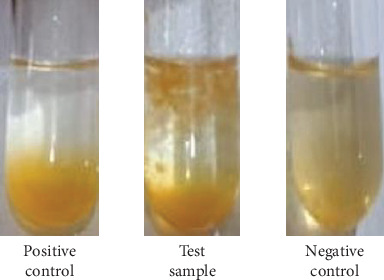
Clarification of orange juice. Positive control, test, and negative control are commercial pectinase, pectinase of *G. candidum* AHC1, and without pectinase, respectively.

The pectinase has been applied for the quality enhancement and clarification of fruit juices [43]. Clarity is an important index of fruit juices [44]. In the present study, crude pectinase from the mutant yeast strain, AHC1, was used to treat the orange juice. The data showed a remarkable increase in orange juice yield from the enzyme-treated juice sample (90.34%) in comparison to the control juice (70.56%) (Table 5). Previously, the use of pectinase from the wild-type *G. candidum* AA15 resulted in only 61% clarification of orange [16]. The result obtained in this study is consistent with the previous research on the juice yield increment after the pectinase treatment of guava, apricot, soursop, raspberry, jujube, and banana [45–50]. This increase in the yield may be attributed to the release of water molecules and soluble solids after breaking polysaccharides by the enzyme during incubation time [51]. Furthermore, the effect of pectinase showed a reduction in the juice turbidity, which could be due to pectin hydrolysis by pectinase (Table 5). Turbidity is a crucial factor for assessing both the stability and sensory quality of fruit juice. Pectin, functioning as a binder, enhances viscosity, which in turn boosts cloudiness and is the main cause of haziness in fruit juice [52]. According to Makebe et al. [48], pectinase breaks down pectin molecules and facilitates the creation of protein–pectin complexes. This process removes colloidal particles from the juice, thereby reducing its turbidity. The clarity of tested fruit juice was significantly improved after treatment with pectinase of the mutant yeast strain, *G. candidum* AHC1 (Figure 5). However, further research is required, particularly to evaluate the sensory attributes of the juice prior to determining the commercial value and safety of the orange juice clarification.

## 4. Conclusion

In this study, three pectinolytic yeasts fermented orange peels through solid-state and submerged fermentation. *G. candidum* AHC1, a mutant strain, yielded higher titers of pectinase (76.08 IU mL^−1^) under solid-state fermentation, and therefore, it was selected for characterization purposes. The CCD approach was used to optimize conditions for pectinase activity. Under optimum conditions of 16.47 min reaction time, 1.89% substrate concentration, pH 5.4, and temperature 35°C, pectinase exhibited 87 IU mL^−1^ activity. Low values of *K*_*m*_ of the purified pectinase from *G. candidum* AHC1 demonstrated the high affinity of the enzyme towards its substrate. An increase in juice yield and clarity of orange juice indicated the degradation of pectin polysaccharide by the pectinase of the mutant yeast strain. Pectinase produced through the fermentation of orange peels and its application for the treatment of orange juice to get a high juice yield with less turbidity indicate the potential of this research to prove the circular bioeconomy approach. However, further studies are required to establish the application of this pectinase in the food industry.

## Figures and Tables

**Table 1:** Central composite design affecting pectinase activity of *G. candidum* AHC1.

**Run order**	**Factors**				
	**Temperature (°C)**	**pH**	**Substrate concentration (%)**	**Reaction time (min)**	**Pectinase activity (IU mL ** ^**−1**^ **)** ^**a**^
1	29.8	4.6	1.3	18	40
2	32.2	4.6	1.3	18	70.1
3	29.8	5.4	1.3	18	44.5
4	32.2	5.4	1.3	18	68.3
5	29.8	4.6	1.7	18	57.4
6	32.2	4.6	1.7	18	56.9
7	29.8	5.4	1.7	18	60
8	32.2	5.4	1.7	18	77.1
9	29.8	4.6	1.3	22	47.4
10	32.2	4.6	1.3	22	67.9
11	29.8	5.4	1.3	22	6.21
12	32.2	5.4	1.3	22	67
13	29.8	4.6	1.7	22	30.8
14	32.2	4.6	1.7	22	57.4
15	29.8	5.4	1.7	22	35.1
16	32.2	5.4	1.7	22	66.4
17	31	5	1.5	20	1.7
18	31	5	1.5	20	1.6
19	31	5	1.5	20	1.2
20	25	5	1.5	20	6.2
21	37	5	1.5	20	15.33
22	31	3	1.5	20	27.6
23	31	7	1.5	20	32
24	31	5	0.5	20	1.7
25	31	5	2.5	20	11
26	31	5	1.5	10	31.9
27	31	5	1.5	30	2.4

^a^Insignificant standard deviation.

**Table 2:** Analysis of variance of central composite design for pectinase activity by *G. candidum* AHC1.

**Source**	**DF**	**Adj. SS**	**Adj. MS**	**F** ** value**	**p** ** value**
Model	15	15452.4	1030.16	4.88	0.006
Blocks	1	8129.7	8129.75	38.53	< 0.001
Linear	4	1980.4	495.09	2.35	0.119
Temperature	1	988.1	988.12	4.68	0.053
pH	1	5.3	5.31	0.03	0.877
Substrate concentration	1	87.9	87.94	0.42	0.532
Reaction time	1	899.0	898.99	4.26	0.063
Square	4	7398.6	1849.64	8.77	0.002
Temperature∗temperature	1	6400.9	6400.91	30.34	< 0.001
pH∗pH	1	7189.0	7188.97	34.07	< 0.001
Substrate concentration∗substrate concentration	1	6224.5	6224.46	29.50	< 0.001
Reaction time∗reaction time	1	6660.0	6660.05	31.57	< 0.001
Two-way interaction	6	1309.9	218.32	1.03	0.453
Temperature∗pH	1	198.0	197.99	0.94	0.353
Temperature∗substrate concentration	1	230.2	230.16	1.09	0.319
Temperature∗reaction time	1	294.8	294.84	1.40	0.262
pH∗substrate concentration	1	356.1	356.11	1.69	0.220
pH∗reaction time	1	184.2	184.17	0.87	0.370
Substrate concentration∗reaction time	1	46.6	46.64	0.22	0.647
Error	11	2320.8	210.98		
Lack-of-fit	9	2320.7	257.85	3683.60	< 0.001
Pure error	2	0.1	0.07		
Total	26				

*Note:* Model summary: *S* = 14.5252; *R*‐sq. = 86.94%; *R*‐sq.(Adj.) = 69.14%; *R*‐sq.(Pred.) = 0.00%.

**Table 3:** Estimated regression coefficients in terms of coded and uncoded units for central composite design analysis.

**Term**	**Coef**	**SE Coef**	**95% CI**	**T** ** value**
Constant	−153.1	32.6	(−224.7, −81.4)	−4.70
Blocks				
1	154.6	24.9	(99.8, 209.4)	6.21
2	−154.6	24.9	(−209.4, −99.8)	−6.21
Temperature	3.87	1.79	(−0.07, 7.80)	2.16
pH	0.28	1.79	(−3.65, 4.22)	0.16
Substrate concentration	1.15	1.79	(−2.78, 5.09)	0.65
Reaction time	−3.69	1.79	(−7.63, 0.24)	−2.06
Temperature∗temperature	12.74	2.31	(7.65, 17.82)	5.51
pH∗pH	13.50	2.31	(8.41, 18.59)	5.84
Substrate concentration∗substrate concentration	12.56	2.31	(7.47, 17.65)	5.43
Reaction time∗reaction time	12.99	2.31	(7.90, 18.08)	5.62
Temperature∗pH	3.52	3.63	(−4.47, 11.51)	0.97
Temperature∗substrate concentration	−3.79	3.63	(−11.79, 4.20)	−1.04
Temperature∗reaction time	4.29	3.63	(−3.70, 12.29)	1.18
pH∗substrate concentration	4.72	3.63	(−3.27, 12.71)	1.30
pH∗reaction time	−3.39	3.63	(−11.39, 4.60)	−0.93
Substrate concentration∗reaction time	−1.71	3.63	(−9.70, 6.29)	−0.47

**Table 4:** Enzyme kinetic parameters (*V*_max_ and *K*_*m*_) of pectinase of *G. candidum* AHC1 and *G. candidum* AA15.

**Enzyme**	**V**_max_ ** (*μ*M min** ^**−1**^ **)** ^**a**^	**K**_**m**_ ** (mg mL** ^**−1**^ **)** ^**a**^
Crude pectinase of *G. candidum* AHC1	6	16
Purified pectinase of *G. candidum* AHC1	2.46	10
Crude pectinase of *G. candidum* AA15	0.91	24.69

^a^Insignificant standard deviation.

**Table 5:** G*. candidum* AHC1 pectinase effect on orange juice yield and turbidity.

**Sample**	**Juice yield (%)** ^**a**^	**Relative turbidity (%)** ^**a**^
Control (without enzyme treatment)	70.56	3.27
Enzyme-treated juice	90.34	0.13

^a^Insignificant standard deviation.

## Data Availability

The data that support the findings of this study are available from the corresponding author upon reasonable request.

## Supporting Information

Supporting Information Additional supporting information can be found online in the Supporting Information section. Figure S1: Contour plot showing the interactive effect of (a) substrate concentration, temperature; (b) reaction time, temperature; (c) substrate concentration, pH; and (d) reaction time, pH. Table S1: Factors and their levels in central composite design.
