# Supplementary material for: Production and Characterization of Pectinase Through Solid-State Fermentation of Orange Peels by a Mutant Yeast Strain
Source: Int J Food Sci. 2025 Mar 7;2025:8853746. doi: 10.1155/ijfo/8853746 (PMC11991808; doi:10.1155/ijfo/8853746)
Supplement: Supporting Information — Additional supporting information can be found online in the Supporting Information section. Figure S1: Contour plot showing the interactive effect of (a) substrate concentration, temperature; (b) reaction time, temperature; (c) substrate concentration, pH; and (d) reaction time, pH. Table S1: Factors and their levels in central composite design. [file 8853746.f1.docx]

**Production and Characterization of Pectinase through Solid State fermentation of Orange Peels by a Mutant Yeast Strain**

Uroosa Ejaz^a^, Asma Hanif^b^, Ahsan Ali Khan^a^, Laiba Jawad^a^, Isha Rasheed^a^, Bushra Noor^a^, Amal S. Alswat^b^ Muhammad Sohail ^c*^

^a^ Department of Biosciences, Faculty of Life Science, SZABIST University, Karachi 75600, Pakistan

^b^Department of Biotechnology, College of Science, Taif University, P.O. Box 11099, Taif 21944, Saudi Arabia

^*^Correspondence: [msohail@uok.edu.pk](mailto:msohail@uok.edu.pk)

**Supplementary File**

**(a)**

**(b)**

**(c)**

**(d)**

**(e)**

**Fig. S1** Contour plot showing the interactive effect of (a) Substrate concentration, Temperature (b) Reaction time, Temperature (c) Substrate concentration, pH (d) Reaction time, pH.

**Table S1** Factors and their levels in central composite design.

| **Factor** | **Level of factors** | | | | |
| --- | --- | --- | --- | --- | --- |
|  | **1** | **2** | **3** | **4** | **5** |
| Temperature (°C) | 25 | 29.8 | 31 | 32.2 | 37 |
| pH | 3 | 4.6 | 5 | 5.4 | 7 |
| Substrate concentration (%) | 0.5 | 1.3 | 1.5 | 1.7 | 2.5 |
| Reaction time (min) | 10 | 18 | 20 | 22 | 30 |
